# Acceptability, Feasibility, and Quality of Telehealth for Adolescent Health Care Delivery During the COVID-19 Pandemic: Cross-sectional Study of Patient and Family Experiences

**DOI:** 10.2196/32708

**Published:** 2021-11-15

**Authors:** Sarah M Wood, Julia Pickel, Alexis W Phillips, Kari Baber, John Chuo, Pegah Maleki, Haley L Faust, Danielle Petsis, Danielle E Apple, Nadia Dowshen, Lisa A Schwartz

**Affiliations:** 1 Division of Adolescent Medicine Children's Hospital of Philadelphia Philadelphia, PA United States; 2 Department of Pediatrics Perelman School of Medicine University of Pennsylvania Philadelphia, PA United States; 3 Wake Forest School of Medicine Winston-Salem, NC United States; 4 Department of Psychiatry Perelman School of Medicine University of Pennsylvania Philadelphia, PA United States; 5 Leonard Davis Institute of Health Economics University of Pennsylvania Philadelphia, PA United States

**Keywords:** telehealth, telemedicine, adolescent, COVID-19, acceptability, feasibility, young adult, teenager, cross-sectional, patient experience, experience, efficiency, equity, survey

## Abstract

**Background:**

Data regarding the acceptability, feasibility, and quality of telehealth among adolescents and young adults (AYA) and their parents and caregivers (caregivers) are lacking.

**Objective:**

The aim of this study was to assess the noninferiority of telehealth versus in-person visits by comparing acceptability with respect to efficiency, effectiveness, equity, patient-centeredness, and confidentiality.

**Methods:**

Cross-sectional web-based surveys were sent to caregivers and AYA following video visits within an Adolescent Medicine subspecialty clinic in May-July 2020. Proportions of AYA and caregivers who rated telehealth as noninferior were compared using chi-squared tests. Feasibility was assessed via items measuring technical difficulties. Deductive thematic analysis using the Institute of Medicine dimensions of health care quality was used to code open-ended question responses.

**Results:**

Survey response rates were 20.5% (55/268) for AYA and 21.8% (123/563) for caregivers. The majority of the respondents were White cisgender females. Most AYA and caregivers rated telehealth as noninferior to in-person visits with respect to confidentiality, communication, medication management, and mental health care. A higher proportion of AYA compared to caregivers found telehealth inferior with respect to confidentiality (11/51, 22% vs 3/118, 2.5%, *P*<.001). One-quarter (14/55) of the AYA patients and 31.7% (39/123) of the caregivers reported technical difficulties. The dominant themes in the qualitative data included advantages of telehealth for efficiency and equity of health care delivery. However, respondents’ concerns included reduced safety and effectiveness of care, particularly for patients with eating disorders, owing to lack of hands-on examinations, collection of vital signs, and laboratory testing.

**Conclusions:**

Telehealth was highly acceptable among AYA and caregivers. Future optimization should include improving privacy, ameliorating technical difficulties, and standardizing at-home methods of obtaining patient data to assure patient safety.

## Introduction

With the rapid shift to video visits during the COVID-19 pandemic, adolescents, who are typically digital natives, have been key consumers of technology-delivered health care [1]. Prior to COVID-19, telehealth was seen as a potential tool to increase access to care and reduce health disparities for adolescents, but geographic restrictions and limited reimbursement led to low utilization [2]. Widespread adoption of telehealth was facilitated by emergency waivers issued by the Centers for Medicare and Medicaid Services, which allowed for geographic flexibility and expanded reimbursement, and the proliferation of Health Insurance Portability and Accountability Act compliant videoconferencing platforms [3,4]. Most commercial insurers quickly followed in relaxing telehealth restrictions to keep pace.

With the rapid transition to telehealth during the COVID-19 pandemic, data gathering of end-user acceptability of telehealth has lagged. The crisis conditions of the pandemic resulted in minimal opportunity for stakeholder input and design from adolescents and young adults (AYA) and their families. Even prior to the pandemic, there were limited data on the acceptability of telehealth for adolescents, and existing studies were mostly confined to mental and sexual health care [5-7], thus neglecting other areas of adolescent health care delivery, including gender-affirming care and management of eating disorders. Although recent systematic reviews demonstrate acceptability of telehealth for a variety of pediatric and adult conditions and modest effect sizes for effectiveness for telemedicine management of pediatric conditions, including asthma, attention deficit hyperactivity disorder, and depression, the acceptability of video-delivered care for a broad sample of adolescent health conditions remains unknown [1,8].

Telehealth for adolescent care presents unique use case challenges. Adolescent Medicine service providers navigate additional confidentiality barriers, frequently need to integrate mental health care into visits, and often practice within interdisciplinary care teams, including psychologists, nutritionists, and social workers. Additional protections are needed to maintain confidentiality during adolescent enrollment within electronic health portals and telehealth applications while still allowing for parent and caregiver (caregiver) proxy access to essential health care information [9]. For example, caution is needed to assure that sensitive test results (such as pregnancy testing) are not released to parents through portals without adolescent consent and that confidential telehealth visits for sexual health services are not “visible” to parents. Early analyses have demonstrated successful adoption of telehealth, with high uptake rates for adolescent health care over periods of just days to weeks [9,10]. However, separate from adoption metrics, acceptability and feasibility assessments are essential to assure that telehealth is delivered with equivalent confidentiality protections to in-person care. The importance of confidential care has been amplified by the pandemic, given the rising rates of mental health conditions that necessitate additional privacy protections for both data collection and treatment delivery [11]. Early data from Adolescent Medicine providers demonstrate challenges to ensuring privacy and confidentiality, despite use of headphones, platform chat functions, and yes/no history-taking questions [12]. Providers have also noted that some patients from lower socioeconomic status households experienced greater difficulty securing private space owing to more crowded living arrangements, thereby presenting a potential challenge to equity [12].

Concerns surrounding widespread implementation of telehealth for adolescents remain, including threats to quality of care across the Institute of Medicine (IOM) dimensions of health care quality: safety, effectiveness, timeliness, efficiency, equity, and patient-centeredness [13,14]. Although telehealth has the potential to increase the reach of health care, early data show that it may paradoxically worsen health disparities owing to differential access to wireless internet, private spaces for visits, and mobile devices across race and socioeconomic status [15,16]. An additional area for concern is patient safety, as the lack of hands-on physical examinations and standardized collection of vital signs could lead to errors in diagnosis [17].

The perspectives of both patients and caregivers are critical for assessing the acceptability of telehealth for adolescents. The American Academy of Pediatrics Supporting Pediatric Research in Outcomes and Utilization of Telehealth (SPROUT) research network developed the SPROUT Telehealth Evaluation and Measurement (STEM) framework, a mechanism to evaluate perspectives on telehealth across stakeholders [18]. The Experience branch of the STEM framework emphasizes the need to understand patient and caregiver perspectives on multiple visit aspects, including overall satisfaction, communication quality, and impact on family routines [18]. We therefore sought to examine patient and caregiver attitudes toward telehealth in an Adolescent Medicine subspecialty clinic system. Our primary aim was to determine the acceptability, feasibility, and quality of telehealth for delivery of adolescent health care among patients and caregivers. A secondary aim sought to evaluate the agreement between patient and caregiver responses on acceptability measures.

## Methods

We conducted a cross-sectional web-based survey to assess attitudes toward telehealth in AYA and parents and caregivers (caregivers).

### Settings and Participants

Participants or their dependents received care within an Adolescent Medicine subspecialty clinic, within a large academic pediatric hospital network in the Philadelphia area. The clinic provides contraceptive and gynecologic services, gender-affirming care, HIV treatment and prevention, and management of eating disorders for AYA. The clinic transitioned from 100% in-person visits to majority synchronous video visits starting March 2020 owing to the COVID-19 pandemic [10]. The telehealth platform allowed for a multiple user interface, and visits were attended by multiple clinical team members, including registered dieticians, social workers, psychologists, and interpreters as needed. Patients aged ≥13 years who completed a video visit from May-July 2020 were eligible for enrollment. Caregivers were eligible if their child <18 years of age completed a video visit during the study period or if they accompanied their child 18 years or older in a video visit (ie, the patient did not attend the visit independently). Patients and caregivers could participate independent of each other, and the data therefore do not represent patient/caregiver dyads. Potential participants were called before their telehealth visit by study staff, had contact information confirmed, and were informed about the survey. After visits were completed, links to research electronic data capture–based surveys were sent via text message or email to the participant and, separately, their caregiver, per inclusion criteria.

### Measures

The 32-item (AYA) and 29-item (caregiver) web-based surveys assessed telehealth acceptability and feasibility. Survey items were adapted from previously validated scales, and items were selected using a modified Delphi procedure with experts from Adolescent Medicine, psychology, and informatics [19]. Telehealth acceptability was measured on a 5-point Likert scale comparing telehealth to in-person care with respect to provider-patient communication, convenience, privacy, and achieving goals of care. Feasibility was assessed via questions regarding technical difficulties with visits. Additional independent measures were included for AYA and caregivers, respectively. AYA surveys included items addressing ability to find a private space for the visit and whether there were opportunities to speak with their provider alone. Caregivers provided both their own and their child’s demographic information and completed an additional question on their perceptions of how well their child’s concerns were addressed at telehealth visits compared to in-person visits. In order to capture additional perspectives on telehealth that may not have been captured in our measures, both surveys contained 3 open-ended questions: (1) what are the disadvantages of telehealth compared to in-person visits? (2) what are the advantages of telehealth compared to in-person visits? and (3) please let us know any additional areas in which you felt telehealth was different from in-person visits.

### Quantitative Analysis

Demographic characteristics of patients and caregivers were assessed via descriptive statistics, including means, medians, and standard deviations. For items comparing in-person to telehealth visits, we assessed noninferiority of telehealth to in-person care by dichotomizing responses into 2 categories: telehealth better or the same as in-person and telehealth worse than in-person. As our primary aim was to assess acceptability for both caregivers and adolescents and to identify areas for optimization to assure joint acceptability, we compared proportions of each population rating telehealth as noninferior to in-person care by using chi-squared and Fisher exact test. All analyses were completed in Stata 15 (College Station, TX, StataCorp LP).

### Qualitative Analysis

Three independent coders qualitatively analyzed responses to the open-ended questions in the survey regarding telehealth advantages and disadvantages. The primary (AWP) and secondary (PM, HLF) coders reviewed the open-text survey responses by using a semiquantitative spreadsheet approach, which captured the descriptions and frequencies of themes. The patient and, separately, caregiver-specific responses were independently double-coded using deductive thematic analysis to identify themes unique to the patient and caregiver experiences. The coding team developed an initial codebook of themes based on consensus with each coder and then they separately applied the codebook themes to the entirety of the open-text survey data. Any coding discrepancies were resolved by consensus. To ground findings within an existing health care quality framework, the primary coder categorized the final themes according to the IOM dimensions of health care quality: safety, effectiveness, timeliness, efficiency, equity, and patient-centeredness [13,14]. All procedures were reviewed and deemed by the Institutional Review Board to be exempt as quality improvement.

## Results

### Quantitative Data

In May-June 2020, 268 and 563 surveys were deployed to unique AYA patients and caregivers, respectively, with a response rate of 20.5% (55/268) for AYA and 21.8% (123/563) for caregivers. The majority of the patient and caregiver respondents were White cisgender females (Table 1). The race and sex distributions of patient survey respondents were representative of the patient population seen by the clinic during spring 2019. The most common visit reasons were eating disorders (18/55, 33% patients, 52/123, 42.3% caregivers) and gynecology/reproductive health (18/55, 33% patients, 44/123, 35.8% caregivers).

The majority of the visits were conducted by physician providers (Table 2). Most AYA and caregivers used a smartphone with a Wi-Fi connection for their telehealth visit. With respect to confidentiality, nearly all AYA (54/55, 98%) were able to identify a private space for their visit (Table 2) and 36 out of 55 AYA (65%) spoke to a provider alone during their telehealth visit (Table 2). Of the 19 AYA who did not speak to their provider alone, 3 (16%) wanted to do so (Table 2).

With regards to acceptability (Table 3), the majority of AYA and caregivers rated telehealth as noninferior to in-person visits with respect to privacy, communication, managing medication questions, and discussing test results, mood, and mental health. A significantly higher proportion of AYA compared to caregivers felt telehealth was inferior to in-person care with respect to privacy (11/51, 22% vs 3/118, 2.5%, respectively, *P*<.001). There were no other significant differences between AYA and caregivers in the acceptability ratings across domains.

With respect to feasibility, 39 out of 123 (31.7%) caregivers and 14 out of 55 AYA (25%) reported technical difficulties with telehealth, including difficulty accessing the patient portal. However, 104 out 123 caregivers (84.5%) and 49 out of 55 AYA (89%) reported that the technology system was easy to use, and 97 out of 123 caregivers (78.8%) and 38 out of 55 AYA (69%) reported that video visits improved efficiency of care, including time saved, compared to in-person visits (Table 2).

**Table 1:** Demographic characteristics of the survey respondents.

Characteristic			Patient survey (n=55)		Caregiver survey (n=123)
Age^a^ (years), median (IQR)			18 (17-20)		48 (44-51)
**Race^a,b^, n (%)**					
	White	42 (76.4)		104 (86.7)	
	Black	9 (16.4)		14 (11.7)	
	Asian	4 (7.3)		1 (0.8)	
	Native American	1 (1.8)		3 (2.5)	
	Other	4 (7.3)		2 (1.7)	
	Latina^a^	7 (12.7)		6 (5)	
**Sex^a^, n (%)**					
	Male	10 (18.2)		7 (5.8)	
	Female	45 (81.8)		113 (94.2)	
**Gender identity^a^, n (%)**					
	Cisgender male	11 (20)		7 (5.8)	
	Cisgender female	31 (56.4)		113 (94.2)	
	Transgender male	7 (12.7)		0	
	Transgender female	2 (3.6)		0	
	Gender queer/nonconforming/nonbinary	4 (7.3)		0	
**Visit reason^b^, n (%)**					
	Eating disorder	18 (32.7)		52 (42.3)	
	Gynecology/contraception	18 (32.7)		44 (35.8)	
	Gender-affirming care	12 (21.8)		27 (22)	
	HIV treatment/prevention	3 (5.5)		0	
	Mental health/substance abuse	3 (5.5)		2 (1.6)	
	Other	4 (7.3)		4 (3.3)	

^a^Data not provided by 3 (2.4%) caregiver survey respondents.

^b^Checkbox question: participants could select more than one category if applicable; therefore, percentages add to >100%.

**Table 2:** Telehealth visit characteristics.

			Patients (n=55), n (%)		Caregivers (n=123), n (%)
Previous Adolescent Medicine visit			41 (74.6)		98 (79.7)
**Visit location**					
	Home	54 (98.2)		123 (100)	
	Other	1 (1.8)		0	
Able to identify private space			54 (98.2)		N/A^a^
**Providers present^b^**					
	Physician	47 (85.5)		91 (74)	
	Nurse practitioner/Physician assistant	6 (10.9)		25 (20.3)	
	Nurse	4 (7.3)		3 (2.4)	
	Psychologist/licensed professional counsellors	2 (3.6)		12 (9.8)	
	Social worker	2 (3.6)		2 (1.6)	
	Physical/occupational therapist	1 (1.8)		2 (1.6)	
	Dietician	5 (9.1)		6 (4.9)	
	Other	1 (1.8)		5 (4.1)	
**Connection type^b^**					
	Wi-Fi	47 (85.5)		101 (82.1)	
	Data	13 (23.6)		35 (28.5)	
**Device used**					
	Tablet	8 (14.5)		26 (21.1)	
	Smartphone	40 (72.7)		74 (60.2)	
	Desktop computer	7 (12.7)		6 (4.9)	
	Laptop computer	0		17 (13.8)	
**Difficulty of video visit use**					
	Difficult	3 (5.5)		10 (8.1)	
	Neutral	3 (5.5)		9 (7.3)	
	Easy	49 (89.1)		104 (84.6)	
**Technical difficulties^b^**					
	No issues	41 (74.5)		84 (68.3)	
	Video never worked/stopped working	1 (1.8)		15 (12.2)	
	Audio never worked/stopped working	5 (9.1)		15 (12.2)	
	Poor audio/video quality	6 (11)		14 (11.4)	
	Resorted to telephone call	2 (3.6)		6 (4.9)	
	Difficulty signing up for or starting the telehealth application	3 (5.5)		11 (8.9)	
**Would participate in a video visit again**					
	Disagree	8 (14.5)		15 (12.2)	
	Neither agree nor disagree	10 (18.2)		8 (6.5)	
	Agree	37 (67.3)		100 (81.3)	
**Frequency of talking to health care provider alone during in-person visits^c^**					
	Never/almost never	6 (14.6)		N/A	
	Occasionally/sometimes	15 (36.6)		N/A	
	Almost every time/every time	20 (48.8)		N/A	
Talked to provider alone in telehealth visit			36 (65.4)		N/A
**Wanted to talk to provider alone^d^**					
	Disagree	7 (36.8)		N/A	
	Neither agree nor disagree	9 (47.4)		N/A	
	Agree	3 (15.8)		N/A	
**Convenience compared to in-person visit**					
	Telehealth took longer	8 (14.5)		14 (11.4)	
	No difference	6 (10.9)		7 (5.7)	
	Telehealth saved time	38 (69)		97 (78.9)	
	Never had an in-person visit	3 (5.5)		5 (4.1)	

^a^N/A: not applicable.

^b^Participants could select more than one category if applicable; therefore, percentages add to >100%.

^c^Answered only by patients who had attended a previous adolescent clinic visit (n=41).

^d^Answered only by patients who did not speak to their provider alone during their clinic visit (n=19).

**Table 3:** Comparison of patient and caregiver acceptability of telehealth.^a^

Acceptability of telehealth, domain			Telehealth visit noninferior to in-person visit, n (%)				*P* value
			Patients (n=55)		Caregivers (n=123)		
**Safety**							
	I felt comfortable with the privacy of the video visit.^b^	40 (78.4)		114 (96.6)		<.001	
**Effectiveness**							
	Obtaining prescription refills^c^	43 (95.6)		82 (98.8)		.28	
	Managing medication side-effects and questions^d^	43 (93.5)		87 (97.8)		.22	
	Discussing test results^e^	42 (97.7)		72 (96)		.54	
	Discussing mental health^f^	39 (78)		99 (89.2)		.05	
	Receiving referrals to other providers^g^	41 (97.6)		74 (98.7)		.59	
**Timeliness/efficiency**							
	The visit was convenient for me^h^	49 (96.1)		117 (99.2)		.22	
**Equity**							
	I felt comfortable with the way my provider communicated with me^i^	50 (98)		113 (96.6)		.52	
**Patient-centeredness**							
	I felt comfortable discussing private topics alone with my health care provider^j^	45 (86.5)		109 (94)		.11	
	I felt comfortable communicating with my health care provider^b^	48 (94.1)		112 (94.9)		.55	
	I felt my provider paid attention to me^b^	49 (96.1)		118 (100)		.09	
	I felt my provider listened to me^b^	50 (98)		116 (98.3)		.66	
	I felt my concerns were addressed^b^	50 (98)		115 (97.5)		.65	

^a^Chi-squared test was used.

^b^Not applicable or no prior in-person visit for 4 (7.3%) patients and 3 (2.4%) caregivers; data missing for 2 (1.6%) caregivers.

^c^Not applicable or no prior in-person visit for 10 (18.2%) patients and 37 (30.1%) caregivers; data missing for 3 (2.4%) caregivers.

^d^Not applicable or no prior in-person visit for 9 (16.4%) patients and 29 (24.6%) caregivers; data missing for 5 (4.1%) caregivers.

^e^Not applicable or no prior in-person visit for 12 (21.8%) patients and 44 (35.8%) caregivers; data missing for 4 (3.3%) caregivers.

^f^Not applicable or no prior in person visit for 5 (9.1%) patients and 9 (7.3%) caregivers; data missing for 3 (2.4%) caregivers.

^g^Not applicable or no prior in-person visit for 13 (23.6%) patients and 45 (36.7%) caregivers; data missing for 3 (2.4%) caregivers.

^h^Not applicable or no prior in-person visit for 3 (5.5%) patients and 3 (2.4%) caregivers; data missing for 1 (1.8%) patient and 2 (1.6%) caregivers.

^i^Not applicable or no prior in-person visit for 4 (7.3%) patients and 3 (2.4%) caregivers; data missing for 3 (2.4%) caregivers.

^j^Not applicable or no prior in-person visit for 3 (5.5%) patients and 5 (4.1%) caregivers; data missing for 2 (1.6%) caregivers.

### Qualitative Data

Nearly half (n=26) of the 55 patients (47%) and 86 of the 123 caregivers (69.9%) completed the open-ended questions. The demographics of the patient and caregiver responses to the open-ended questions were reflective of the total survey population. The sample was largely White (19/26, 73% AYA; 75/86, 87% of caregivers) cisgender females (19/26, 73% AYA; 75/86, 87% of caregivers). Emergent themes within the IOM quality framework and exemplar quotes are shown in Table 4.

The most frequently cited advantage of telehealth compared to in-person visits was within the IOM dimension of *Timeliness*. Both patients and caregivers indicated that time was saved from no commute or in-person waiting room time and reported financial savings from less work missed and no transportation costs. The second most common theme was “Improved access to care for vulnerable populations” within the *Equity* IOM domain. Patients and caregivers described telehealth as expanding access to people who may experience a variety of challenges with attending in-person Adolescent Medicine visits. Patients also discussed how telehealth improved equity in care delivery, including, but not limited to, reducing misgendering patients by clinic staff.

**Table 4:** Advantages/disadvantages reported in the patient and caregiver open-ended survey responses.

Construct, advantage/disadvantage			Themes		Frequency^a^		Exemplar quotes
**Safety: Delivering health care that minimizes risks and harm, including avoiding preventable injuries and reducing medical errors**							
	Advantage	Improved patient safety		8		…*There’s a greater risk of getting COVID-19 when you do in-person visits rather than telehealth visits.* [Patient]	
	Disadvantage	Increased safety risks due to lack of hands-on data		4		…*I feel that there is potential for parents to miss or overlook clues about teen eating disorders using telemedicine as a primary treatment option.* [Caregiver]	
	Disadvantage	Decreased visit privacy		10		…*Due to my answer-giving at home, I feel it’s not as safe because I have neighbors… and our house is connected to someone else’s house.* [Patient]	
**Effectiveness: Providing services based on scientific knowledge and evidence-based guidelines**							
	Advantage	Improving adherence to treatment recommendations		4		…*Accountability for seeing the doctor has been a powerful motivator for our family to do the right thing.* [Caregiver]	
	Disadvantage	Limited scope of practice		47		…*Telehealth is not able to easily address physical problems, can’t take blood pressure etc*. [Patient]	
**Timeliness: Reducing delays in providing and receiving health care**							
	Advantage	Allowed continuity of care during the pandemic		12		…*I would not prefer [telehealth] as a matter of course but appreciated this visit since there was no other alternative at the moment.* [Caregiver]	
	Advantage	Reduced delays in care		16		…*I know my child will be seen sooner vs coming in after waiting months for appointments due to heavy schedules.* [Caregiver]	
	Disadvantage	Disrupted care due to technical issues		14		…*After checking in, and downloading applications, application repeatedly restarted, only worked for audio … cut out 3 different times and required Doctor/us to switch to a phone call* [Caregiver]	
	Disadvantage	Visit workflow challenges		2		…*I was not made aware that the staff would call my home phone…I specifically asked them not to call, and the call disturbed…my family who were busy with online job interviews and standardized tests.* [Patient]	
**Efficiency: Delivering health care in a manner that maximizes resource use and avoids waste**							
	Advantage	Improved convenience for families		102		…*Usually an appointment …takes us 4 hours and this only took 1 hour for the actual appointment.* [Caregiver]	
	Advantage	Decreased cost to families		9		…*So much cheaper than paying gas tolls and parking plus saves two hours of drive time.* [Caregiver]	
	Disadvantage	Increased financial burden on families		1		…*My daughter does n’t get weighed in or her vitals taken. We have to go to her primary for weight check and [orthostatic vital signs] … which means I pay for a second doc visit.* [Caregiver]	
**Equity: Delivering health care that does not differ in quality according to personal characteristics**							
	Advantage	Improved quality of care for vulnerable populations		45		…*The front desk staff cant misgender me because I don’t interact with them.* [Patient]	
	Advantage	Increased access to care vulnerable populations		7		…*For people who are sick or nonmobile. these visits benefits [them] because they could still get the treatment they need right from home…* [Patient]	
	Disadvantage	Limited resources or technology access impede care		2		…*[Telehealth] may be hard for some people to use or have access to.* [Patient]	
**Patient-centeredness: Providing care that takes into account the preferences and aspirations of individual service users**							
	Advantage	Improved person-centered communication		16		…*I loved it! Doctor was engaged and it felt like a regular visit … I felt like it was less intimidating.* [Patient]	
	Advantage	Strengthened preexisting patient-provider relationships		5		…*We have already met with the doctor and are very comfortable with telehealth appointments.* [Caregiver]	
	Disadvantage	Environmental distractions may impede care		6		…*My family is extremely nosey and it was hard to find a quiet/safe place in my house.* [Patient]	
	Disadvantage	Diminished clinician-patient communication and rapport		28		…*My daughter [was] able to leave the room if not wanting to engage, where in person visits are more engaging.* [Caregiver]	

^a^Frequency of the coded theme among adolescents and young adults and caregivers.

With respect to the disadvantages of telehealth, the most common theme was “Limitations in scope of practice” within the *Effectiveness* IOM domain. Patients and caregivers discussed that the lack of hands-on physical examination and laboratory testing, which were felt to be essential for the delivery of evidence-based care, could lead to decreased quality of care. Patients and caregivers also frequently endorsed challenges to *patient-centeredness*, particularly in communication and building rapport. With respect to *Equity*, one caregiver described the financial burden of telehealth owing to the challenges with a limited scope of practice, where caregivers may be required to pay for separately for both a telehealth visit and an in-person laboratory visit to meet the health needs of their child.

Caregiver responses differed qualitatively from patient open-ended responses in 2 ways. First, caregivers placed greater emphasis on the importance of preexisting provider-patient relationships in successfully creating a comfortable visit environment via telehealth. Second, caregivers more commonly framed telehealth as advantageous with respect to *patient-centeredness*, including comfort, provider-patient communication, and engagement in visits.

## Discussion

### Principal Findings

Within an Adolescent Medicine clinic, we found high acceptability of telehealth among both patients and caregivers. The majority of the patients and caregivers reported that telehealth visits were easy to use and saved time and they expressed willingness to participate in another telehealth visit. Key areas for optimization in telehealth implementation included improving technical problems, which may limit uptake, and ensuring adequate confidentiality standards for AYA in the video visit setting. Although >85% of respondents found the telehealth system easy to use, a quarter of the patients and nearly a third of caregivers reported experiencing at least one technical issue during their telehealth visit. The most common issue across both groups was malfunctioning of the audio component in the video visit. The analysis of telehealth satisfaction among pediatric neurology service providers during the COVID-19 pandemic similarly revealed high levels of satisfaction, despite nearly 40% encountering technical challenges, and the providers surveyed also reported that audio problems were the most common [16]. In order to optimize telehealth quality, it will be essential to resolve technical issues impacting communication of clinical information and treatment recommendations. As technology continues to rapidly evolve, health systems likely need to “go back to the drawing board” to conduct more extensive usability testing on their systems. The user-centered design process is typically part of scale-up of new mobile health interventions but was bypassed owing to the urgency of the pandemic. Periods of respite between COVID surges may provide an opportunity to refine the user experience. Lastly, as health systems optimize their technology, consideration should be given to integrating remote patient monitoring options such as heart rate monitors, actigraphy, and pulse oximetry to augment video history and examination findings [20-22]. These digital tools also hold promise as health-promoting interventions in their own right. Remote patient monitoring strategies with real-time patient feedback may improve disease self-management and treatment adherence in conditions such as asthma and diabetes for adolescents.

Privacy was the only acceptability measure in which we found divergence between caregivers and adolescents. A significantly higher proportion of patients rated telehealth as inferior to in-person care for privacy. This finding suggests that AYA perceptions of visit privacy may be more complex than the simple ability to identify a private space for the visit, which >98% of patients were able to do. Prior research efforts with Adolescent Medicine providers have identified several strategies for optimizing privacy and confidentiality during telehealth visits. For at-home visits in which patients have access to adequate technology and space for the visit, these include the use of headphones, yes/no history-taking questions, use of chat functions, and using background white noise to lessen the chance that others in the household will overhear [12,23]. In efforts to improve telehealth privacy, special attention should be paid to adolescents who lack stable housing, private space, or consistent access to technology. These include creating dedicated patient telehealth “drop-in” kiosks stocked with computers or tablets and soundproof space at essential locations that may remain open in a public health crisis, such as pharmacies, primary care clinics, or schools. In addition, models from the Veterans Administration have demonstrated that delivery of tablets to unstably housed individuals is a feasible strategy for maintaining access to telehealth for vulnerable populations [12,15,23-25].

The high acceptability and convenience of telehealth reported by AYA patients and caregivers point to potential benefits of integrating telehealth visits in adolescent care in the future years. However, the future of telehealth in the United States remains uncertain. In April 2021 and July 2021, the US Department of Health and Human Services renewed the declaration of the COVID-19 pandemic as a public health emergency for an additional 90 days [26]. Under this renewal, the blanket waivers issued by the Centers for Medicare and Medicaid Services to increase geographic flexibility and expanded reimbursement remained in effect [3]. In the absence of a further renewal, however, many of these waivers may no longer apply, making telehealth far less feasible. Some commercial insurers began withdrawing additional provisions, allowing for expanded telehealth reimbursement in fall 2020, with more following in winter and spring 2021. Given broad state discretion, telehealth policy for Medicaid and Children’s Health Insurance Policy is also in flux across states. High acceptability of telehealth suggests that the integration of telehealth as an additional care delivery mode may be highly beneficial. In addition, given the increasing rates of adolescent mental health diagnoses, suicidal ideation, and suicide attempts during the pandemic [27-29], telemedicine will be an essential means of delivering evidence-based mental health care to youth, given the dearth of available in-person services [1]. Whether our health system can rise to this challenge will depend on the continuation of policies that, by lessening geographic restrictions and achieving parity with in-person visit reimbursement rates, enabled widespread telehealth use.

Our analysis has several limitations. The survey response rate was low, and therefore, may not provide a complete picture of patient and caregiver experiences with telehealth. Surveys were sent to patients attending visits during May-June 2020, when COVID-19 cases were rapidly rising in the United States. Many patients and caregivers were experiencing abrupt changes to their routines and additional stressors during these months, which may have limited the response rate. However, the response rate of the patients compared to that of the caregivers was approximately the same, and the patient race and sex demographic distribution did not differ significantly from patients seen in the clinic for in-person visits during spring 2019. The majority of the respondents were White, non-Hispanic, cisgender females, and therefore, our results may not be generalizable to other populations. Previous analyses of telehealth during COVID-19 in both pediatric and adult populations have demonstrated racial and socioeconomic disparities in telehealth utilization, with non-White patients, Latinx patients, and patients with low median household incomes having both lower overall utilization and utilizing audio only visits more often than audio plus video [16,30-32]. These studies provide an early signal that rapid introduction of telehealth, in many instances, has led to the unintended consequence of widening the equity gap in health care delivery. Our telehealth platform was designed to allow multiple users, including interpreters, to attend visits. This multiuser interface may not be generalizable to less-resourced health systems, and thus, attention should be paid in future research to capture the experiences of populations with limited English proficiency in a diversity of health systems. Understanding and addressing emerging health disparities and evaluating telehealth acceptability among marginalized groups will be crucial in any future implementation of telehealth.

### Conclusions

Widespread telehealth adoption in response to the COVID-19 pandemic altered health care delivery during 2020 and 2021. We demonstrate high acceptability of telehealth by AYA and caregivers of AYA, a population for which very little was previously known about the acceptability and feasibility of the use of telehealth. Our data support the importance of maintaining reimbursements for telehealth as a strategy for adolescent health care delivery. Future research addressing telehealth in adolescents should focus on ensuring equity, optimizing the end-user experience, and improving confidentiality protections.

## Abbreviations

AYA: adolescents and young adults
IOM: Institute of Medicine
SPROUT: Supporting Pediatric Research in Outcomes and Utilization of Telehealth
STEM: SPROUT Telehealth Evaluation and Measurement
